# Supplementary material for: FOXE1 Gene Dosage Affects Thyroid Cancer Histology and Differentiation In Vivo
Source: Int J Mol Sci. 2020 Dec 22;22(1):25. doi: 10.3390/ijms22010025 (PMC7792778; doi:10.3390/ijms22010025)
Supplement: Supplementary file 1 [file ijms-22-00025-s001.pdf]

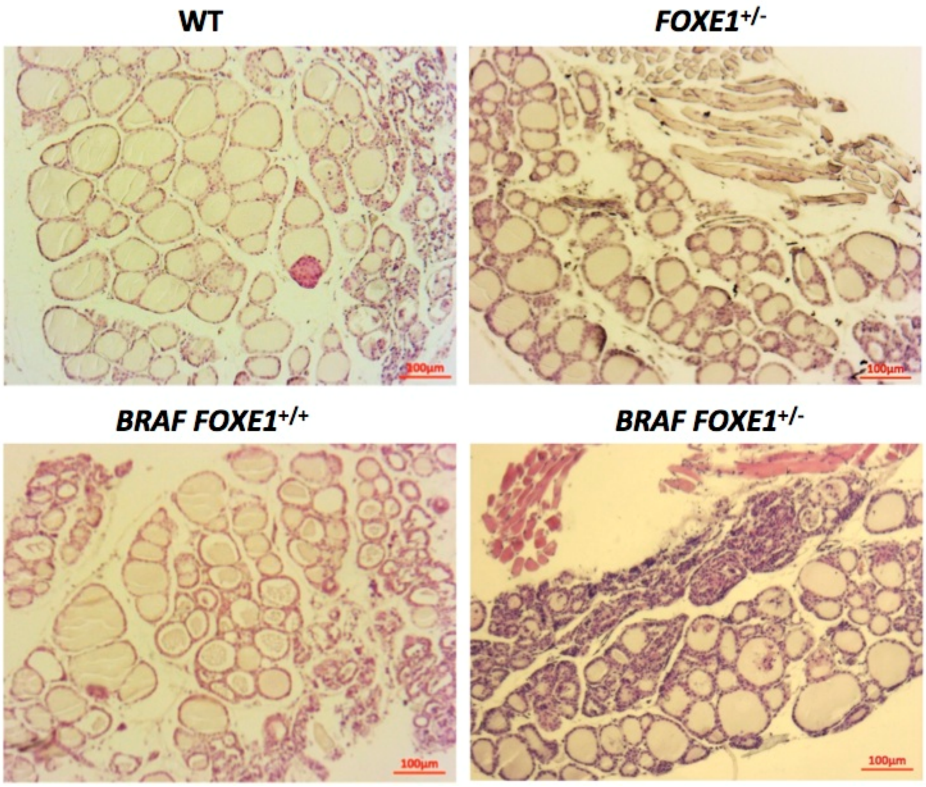

Figure S1. Hematoxylin and Eosin staining of 7 µm sections of thyroids from untreated WT, *FOXE1*$^{+/-}$, *BRAF FOXE1*$^{+/+}$ and *BRAF FOXE1*$^{+/-}$ mice. Each image is representative of six different images.

A

Genotype	No. of mice per treatment	
	**NT**	**+DOX**
**WT**	6	6
FOXE1$^{+/-}$	6	6
BRAF FOXE1$^{+/+}$	6	6
BRAF FOXE1$^{+/-}$	6	6

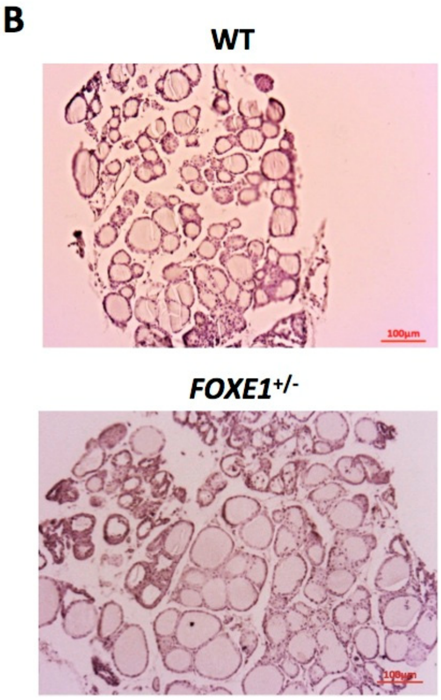

Figure S2. (**A**) Twelve mice of each useful genotype were divided in two experimental groups of six animals per group: the untreated group (NT) and the group fed with a 2500 mg/kg doxycycline supplemented fodder for one week (+DOX). (**B**). Hematoxylin and Eosin staining of 7 µm sections of WT and *FOXE1*$^{+/-}$ thyroid after one week of doxycycline administration. 10X magnification is shown. Each image is representative of six different images.

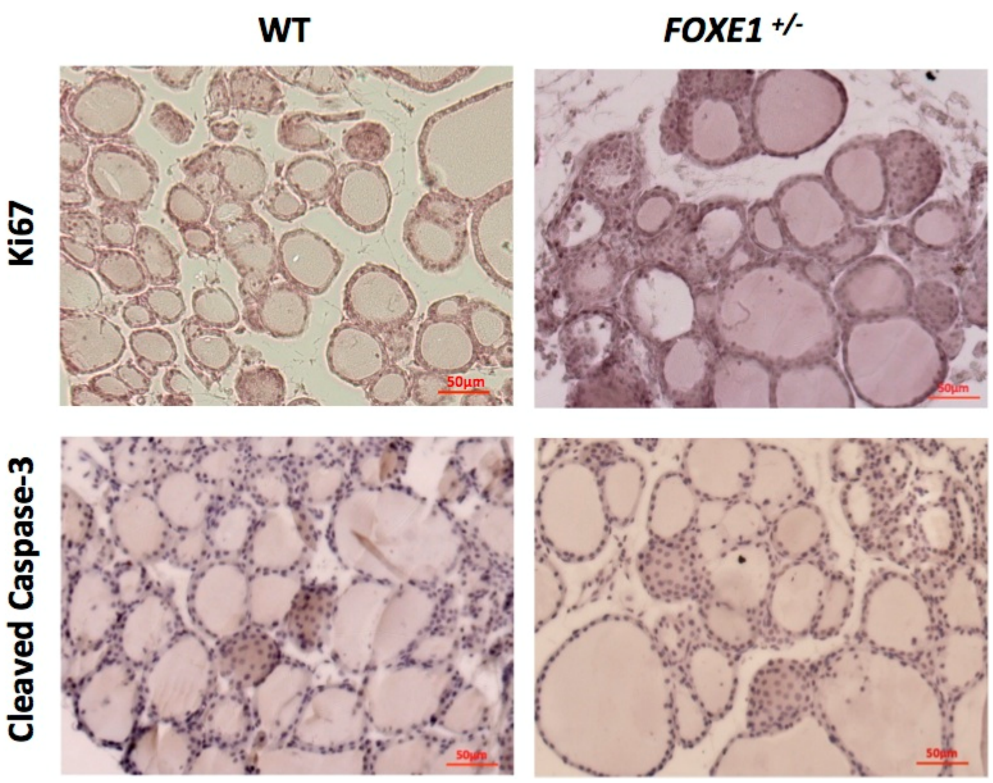

Figure S3. IHC staining for Ki67 (upper panel) and Cleaved Caspase-3 (bottom panel) was performed on 7 µm section of thyroids from WT and *FOXE1*$^{+/-}$ mice after one week of doxycycline treatment. Hematoxylin staining of nuclei was then performed. 20X magnifications are shown. Each image is representative of six different images.

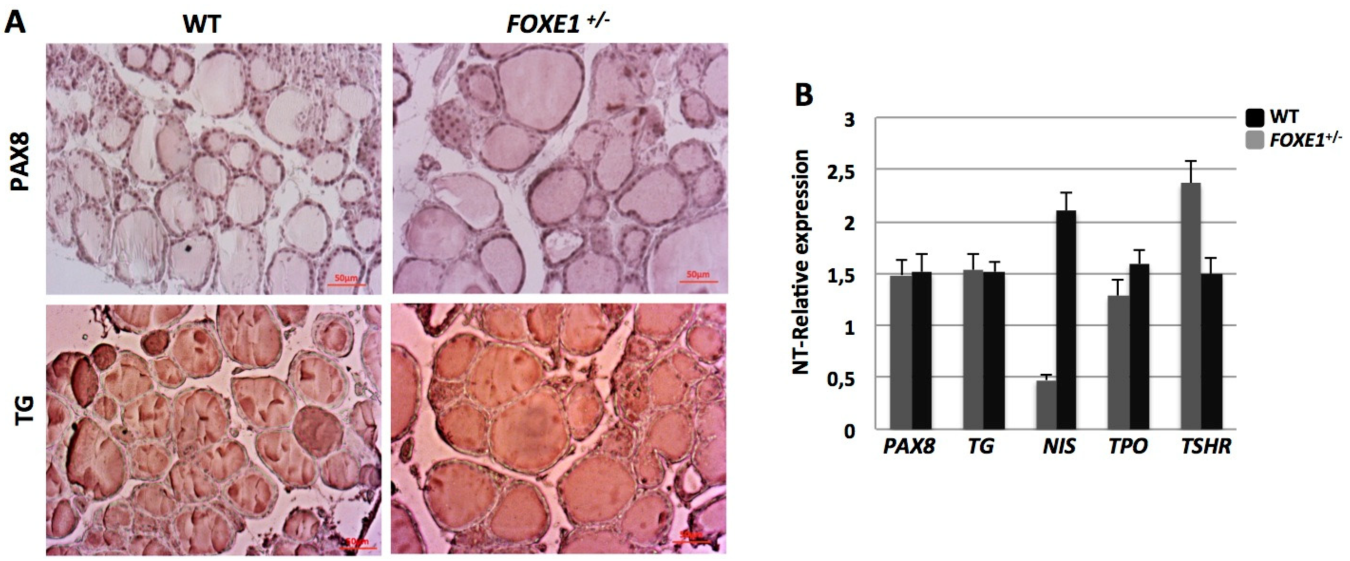

Figure S4. (**A**). IHC staining for PAX8 (upper panel) and TG (bottom panel) was performed on 7 μm section of thyroids from WT and *FOXE1*$^{+/-}$ mice after one week of doxycycline treatment. 20X magnifications are shown. Each image is representative of six different images. (**B**). Total RNA was extracted from pooled thyroids of six WT and six *FOXE1*$^{+/-}$ mice, either treated and untreated (NT). Differentiation markers were analyzed by quantitative RT-PCR. Results are normalized using β-actin and reported as the fold change ($2^{-\Delta\Delta C_t}$) of WT and *FOXE1*$^{+/-}$ treated mice respect to the untreated mice with same genotype. Means ± SD are shown.
